# Differential Expression of PD-L1 in Central and Peripheral and TTF1-Positive and -Negative Small-Cell Lung Cancer

**DOI:** 10.3389/fmed.2020.621838

**Published:** 2021-01-25

**Authors:** Shili Yu, Meng Jia, Yuemin Li, Ping-Li Sun, Hongwen Gao

**Affiliations:** Department of Pathology, The Second Hospital of Jilin University, Changchun, China

**Keywords:** small-cell lung cancer, PD-L1, central and peripheral, immunohistochemistry, clone 22C3, TTF-1

## Abstract

**Background:** Central and peripheral location as well as thyroid transcription factor-I (TTF-1) expression was reported to be associated with different characteristics and prognosis of small-cell lung cancer (SCLC). This study aimed to investigate differential expression of PD-L1 in different SCLC subtypes, and in biopsy and resection specimens.

**Methods:** We retrospectively analyzed 142 SCLC tumor samples using immunohistochemistry to correlate PD-L1 (22C3) expression with clinicopathologic features and survival data.

**Results:** PD-L1 expression was found in 19.7% SCLCs (28/142) and was more frequent in females than in males (32%, 16/50 vs. 13%, 12/92, *p* = 0.009), in central type than in peripheral type SCLCs (26%, 26/100 vs. 4.8%, 2/42, *p* = 0.003), and in TTF-1 positive than in negative SCLCs (23.8%, 25/105 vs. 8.1%, 3/37, *p* = 0.039). PD-L1 expression was associated with vascular (*p* = 0.001) and lymphatic invasion (*p* = 0.001). There was no significant difference in PD-L1 expression between biopsy and resection specimens. On univariate analysis, patients with PD-L1 expression had significantly shorter progression-free survival (PFS; *p* = 0.026) and overall survival (OS; *p* = 0.012). Multivariate analysis revealed that PD-L1 expression was an independent prognostic factor for OS (HR, 2.317; 95% CI 1.199–4.478; *p* = 0.012) and PFS (HR, 1.636; 95% CI 0.990–2.703; *p* = 0.051) in SCLC.

**Conclusions:** PD-L1 expression was more frequent in central type, TTF-1 positive SCLCs, and predicted a poor clinical outcome in these patients. Therefore, tumor location and TTF-1 expression could predict expression status of PD-L1, and could potentially serve as clinical response to immunotherapy.

## Introduction

Small-cell lung cancer (SCLC) is an aggressive malignant disease with early development of metastasis. Surgery generally benefits only about 1% of the patients (1). The first-line therapy for SCLC is platinum-based systemic chemotherapy which has a good initial response rate. However, the vast majority of patients relapse and become resistant within a year (2). Immunotherapy, especially the one based on programmed death 1/programmed death-ligand 1 (PD-1/PD-L1) checkpoint inhibitor has generated significant breakthrough in the treatment of SCLC recently (3, 4).

The expression of PD-L1 on tumor cells has demonstrated good correlation with response in some clinical trials, but positive responses were only observed in a relatively small section of the patients with PD-L1 expression. Moreover, evidence suggests that patients with PD-L1–negative tumors may also respond to immunotherapy (2, 5, 6). This has prompted many researchers to seek out the subtype of patients who could benefit from immune treatments that has not yet been identified in SCLC.

The morphological features of small-cell carcinoma are relatively consistent between different tumors and different areas of an individual tumor. However, studies have shown inter-and intratumoral heterogeneity of SCLC, and the impact of these differences on the response to specific therapeutic agents in both patients and animal models (7–10). SCLC tumors have been grouped into distinct subgroups based on a few factors including tumor location, expression of immunohistochemical markers, and genetic profiles. Previous evidence suggest that the central and peripheral type SCLCs show different immunohistochemical phenotypes (TTF-1 and some neuroendocrine markers) and prognosis (11, 12). In a mouse model of SCLC, central and peripheral tumors showed different response to cisplatin treatment (7). TTF-1 was expressed in 81–97% of SCLCs and TTF-1–negative SCLCs exhibited decreased neuroendocrine differentiation, but the prognosis and treatment efficacy of its expression remains unclear because of the limited number of reports (8, 11, 13). These facts raise the questions as to whether PD-L1 expression differs in different types of SCLC and what may be the prognostic significance of its expression?

Although few studies have evaluated the expression of PD-L1 and its prognostic role in SCLC, the results are inconsistent (14, 15). No study has evaluated PD-L1 expression in different SCLCs based on tumor location, TTF-1 expression, and specimen types so far. Therefore, to address the above questions, we investigated the expression of PD-L1 in different types of SCLCs in terms of clinicopathologic characteristics and prognosis in this study, with the objective of finding more specific candidates for immunotherapy.

## Materials and Methods

### Patients and Data Collection

In this study, a total of 2,524 SCLC patients (24%, including 71 resection cases) were selected from 10,458 lung cancer patients who were admitted at The Second Hospital of Jilin University (Jilin, China) between January, 2009 and December, 2018. A total of 142 SCLC patients with follow-up data were finally selected. Patients who had received preoperative neoadjuvant chemotherapy or radiotherapy were excluded in this study. A total of 54 resection cases with medical record were enrolled in this study. The median follow-up period was 21.3 months. The overall 3-year survival rate of the patients was 10.8%, and 5-year survival was 0%. Smoking status was obtained from the electronic medical records or through telephone surveys. Tumors involving segmental or more proximal bronchi were defined as central type, whereas tumors involving subsegmental or more distal bronchi were defined as peripheral type. A few cases were excluded from the study because of the difficulty in grouping them into the central or peripheral types. This study was approved by the Ethics Committee of The Second Hospital of Jilin University.

### Definition of Central- and Peripheral-Type SCLC

Definition of central-and peripheral-type SCLC based on previous reports (8, 16, 17), primary tumors involving segmental or more proximal bronchi were defined as central-type tumors. Primary tumors involving subsegmental or more distal bronchi were defined as peripheral-type tumors.

### Immunohistochemistry (IHC)

Immunohistochemistry was performed on formalin-fixed, paraffin-embedded tissue sections using the FDA (Food and Drug Administration) and NMPA (National Medical Products Administration) approved PD-L1 IHC 22C3 pharmDx kit on the Dako Autostainer Link 48 platform according to manufacturer recommendations. PD-L1 positivity was defined as membranous staining of any intensity in at least 1% of the tumor cells and inflammatory cells (tumor associated lymphocytes and macrophages) with more than 1% membrane and/or cytoplasm staining of any intensity was defined as positive. The slides were examined independently by two observers (P-LS and SY) blinded to both the clinical and pathologic data.

### Statistical Analysis

All statistical analyses were performed using SPSS version 22.0 for Windows (Inc., Chicago, IL). Categorical variables were compared using Fisher's exact test or Chi-square test. Kaplan-Meier analysis was performed for survival curves, and statistical significance was assessed by the log rank test. To evaluate whether a biomarker was an independent prognostic factor of OS, multivariate analysis using the Cox proportional hazard regression model was performed. All tests were two sided, and *p* < 0.05 was considered statistically significant.

## Results

### Patient Characteristics

The clinicopathologic features of the 142 patients are summarized in Table 1. The cohort consisted of 92 (64.8%) men and 50 (35.2%) women with a median age of 61.72 years (range, 41–87), and included 102 (71.8%) smokers and 40 (28.2%) non-smokers. Of the 142 tumor samples obtained, 88 were biopsy and 54 were resection samples, and among these 86 (60.6%) were limited-stage and 56 (39.4%) were extensive-stage cases. Further, 26.8% (38/142) of the patients presented with distant metastatic disease at the time of diagnosis, and the metastatic sites included supraclavicular lymph node, liver, and contralateral lungs among others. Of the 54 resected SCLCs, eight cases were available with paired preoperative bronchoscopic biopsy specimens.

**Table 1:** PD-L1 status in 142 small-cell lung cancer according to clinicopathologic characteristics.

	**PD-L1 expression status**			
**Variable**	**No. (%)**	**Positive** **No. (%)**	**Negative** **No. (%)**	***P-*value**
Total	142	28 (19.7)	114 (80.3)	
Age (year)				
<60	67 (47.2)	16 (23.9)	51 (76.1)	0.293
≥60	75 (52.8)	12 (16.0)	63 (84.0)	
Sex				
Male	92 (64.8)	12 (13.0)	80 (87.0)	**0.009**
Female	50 (35.2)	16 (32.0)	34 (68.0)	
Smoking status				
Smoker	102 (71.8)	20 (19.6)	82 (80.4)	0.958
Non-smoker	40 (28.2)	8 (20.0)	32 (80.0)	
Specimen types				
Biopsy	88 (62.0)	17 (19.3)	71 (80.7)	1.000
Resection	54 (38.0)	11 (20.4)	43 (79.6)	
Tumor locations				
Central-type	100 (70.4)	26 (26.0)	74 (74.0)	**0.003**
Peripheral-type	42 (29.6)	2 (4.8)	40 (95.2)	
Stage				
Limited-stage	86 (60.6)	17 (19.8)	69 (80.2)	0.985
Extensive-stage	56 (39.4)	11 (19.6)	45 (80.4)	
Pleural invasion				
Present	13 (24.1)	2 (15.4)	11 (84.6)	1.000
Absent	41 (75.9)	9 (22.0)	32 (78.0)	
Vascular invasion				
Present	24 (44.4)	10 (41.7)	14 (58.3)	**0.001**
Absent	30 (55.6)	1 (3.3)	29 (96.7)	
Lymphatic invasion				
Present	20 (37.0)	9 (45.0)	11 (55.0)	**0.001**
Absent	34 (63.0)	2 (5.9)	32 (94.1)	
TTF-1 expression				
Positive	105 (73.9)	25 (23.8)	80 (76.2)	**0.039**
Negative	37 (26.1)	3 (8.1)	34 (91.9)	
Chromogranin A				
Positive	115 (82.1)	26 (22.6)	89 (77.4)	0.165
Negative	25 (17.9)	2 (8.0)	23 (92.0)	
Synaptophysin				
Positive	129 (92.1)	26 (20.2)	103 (79.8)	1.000
Negative	11 (7.9)	2 (18.2)	9 (81.8)	
CD56				
Positive	129 (93.5)	26 (20.2)	103 (79.8)	
Negative	9 (6.5)	2 (22.2)	7 (77.8)	1.000

*The bold values indicates significantly difference*.

Regarding the tumor location, 100 cases (70.4%) were of the central type and 42 (29.6%) cases were of the peripheral type. TTF-1 was expressed in 73.9% (105/142) of the SCLC cases. Moreover, 72.0% (72/100) of the central type tumors and 78.6% (33/42) of the peripheral type tumors showed positivity for TTF-1. There was no significant correlation between TTF-1 expression and tumor type. Most of the cases showed positive expression of the common neuroendocrine markers including chromogranin A (82.1%, 115/140), synaptophysin (92.1%, 129/140), and CD56 (93.5%, 129/138). The Ki-67 labeling index was high (>70%) in almost all cases (99.3%, 141/142).

### Expression and Clinicopathologic Correlation of PD-L1 in Total SCLC

**Figure 1:**
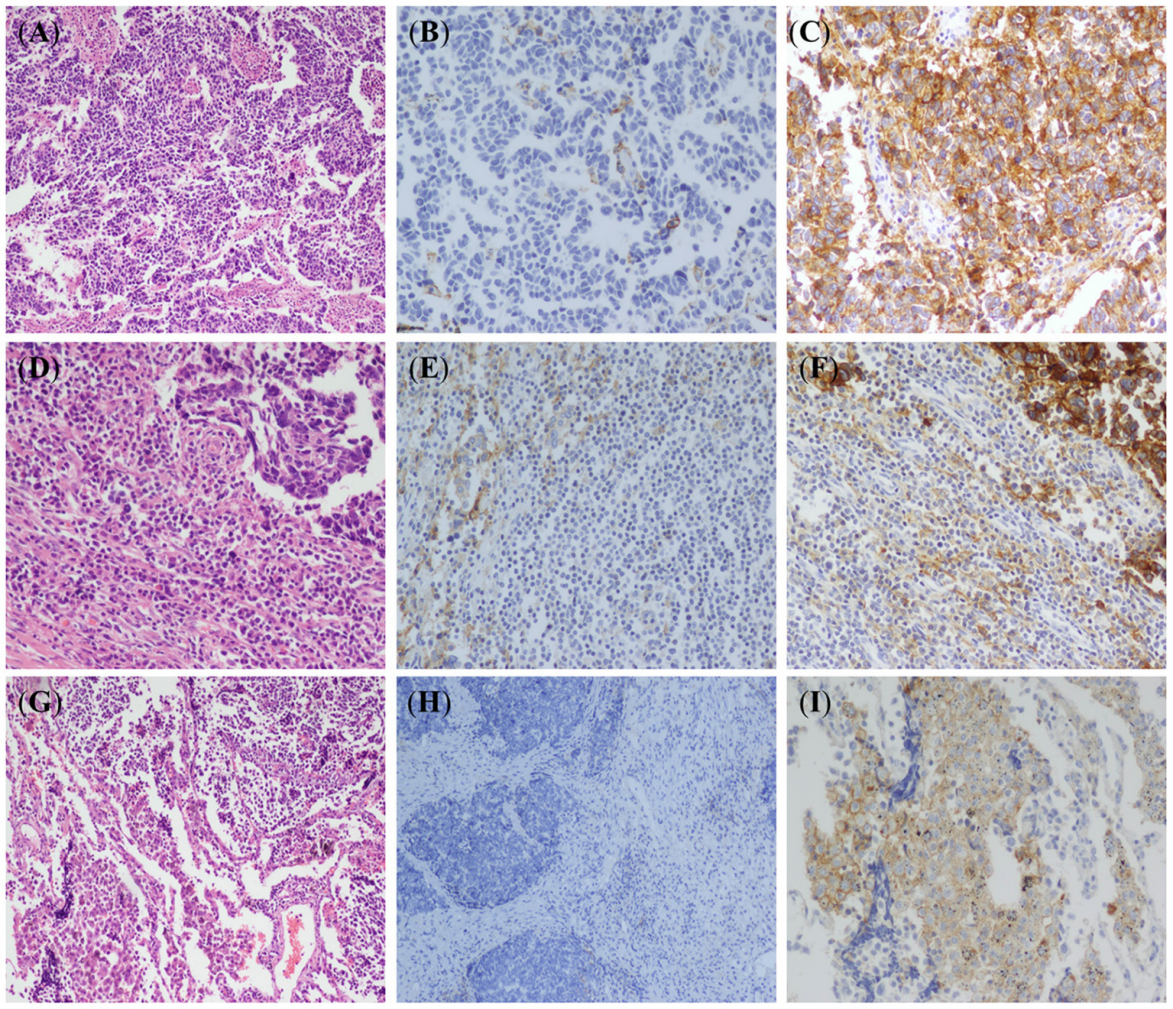
Hematoxylin-eosin (HE) staining and PD-L1 immunohistochemical (IHC) staining in small-cell lung cancer (SCLC). **(A)** HE stained images of SCLC tumor (×100). **(B)** IHC stained images of PD-L1 negative in SCLC tumor cells (EnVision, ×100). **(C)** IHC stained images of PD-L1 expression in SCLC tumor cells (EnVision, ×200). **(D)** Lymphocyte infiltration around tumor cells (HE, ×100). **(E)** IHC stained images of PD-L1 negative in tumor infiltrating lymphocytes (EnVision, ×100). **(F)** IHC stained images of PD-L1 positive cell membranes of tumor infiltrating lymphocytes (EnVision, ×200). **(G)** Macrophages infiltration around tumor cells (HE, ×100). **(H)** IHC stained images of PD-L1 negative in tumor-associated macrophages (EnVision, ×100). **(I)** IHC stained images of PD-L1 positive cell membranes of tumor-associated macrophages (EnVision, ×200).

PD-L1 protein expression was not detected in peribronchial mucus glands and bronchial epithelium, non-neoplastic type I pneumocytes, type II pneumocytes, and mesenchymal cells. Some inflammatory cells in the lung cancer microenvironment including T cells, macrophages, and mast cells showed weak to moderate PD-L1 cytoplasmic and membrane immunoreactivity (Figures 1A–F). PD-L1 expression was detected in the membrane and/or cytoplasm of tumor cells (Figures 1G–I). Tumor cells with more than 1% membrane staining of any intensity was defined as positive and a total of 19.7% (28 of 142) of the SCLC cases were found to express PD-L1. Tumor associated lymphocytes and macrophages with more than 1% membrane and/or cytoplasm staining of any intensity was defined as positive and the total percentage is 41.5% (59/142). The intensity of staining ranged from weak to moderate and strong, and majority of the cases showed moderate staining. The stained tumor percentage of the positive cases was between 2 and 35% with a mean value of 16%.

Among the 142 patients, PD-L1 expression was more commonly observed in females than in males (32%, 16/50 vs. 13%, 12/92, *p* = 0.009), in central type than in peripheral type SCLC (26%, 26/100 vs. 4.8%, 2/42, *p* = 0.003), and in TTF-1 positive than in negative cases (23.8%, 25/105 vs. 8.1%, 3/37, *p* = 0.039). There was a significant correlation between PD-L1 expression and the presence of vascular (*p* = 0.001) and lymphatic (*p* = 0.001) invasion. No significant difference of PD-L1 expression was observed between smokers (19.6%, 20/102) and non-smokers (20.0%, 8/40), biopsy (19.3%, 17/88) and resected specimens (20.4%, 11/54), limited-stage (19.8%, 17/86), and extensive-stage cases (19.6%, 11/56). Correlation between PD-L1 expression and neuroendocrine markers was also evaluated and no correlation was found between PD-L1 expression and any of the neuroendocrine markers (Table 1).

### PD-L1 Expression in Different Types of SCLCs

To investigate whether PD-L1 was differentially expressed in different types of SCLCs, we analyzed the association of PD-L1 expression and some clinicopathological parameters including tumor location (central and peripheral), TTF-1 expression (positive and negative), and sample types (biopsy and resection).

There was a significant correlation between PD-L1 expression and female sex (*p* = 0.035), the presence of vascular invasion (*p* < 0.001), the presence of lymphatic invasion (*p* = 0.004), and TTF-1 expression (*p* = 0.001) in central type SCLC. There were no significant correlations between PD-L1 expression and the clinicopathologic characteristics except a negative correlation with TTF-1 expression (*p* = 0.042) in the peripheral type SCLC (Table 2).

**Table 2:** Correlation between PD-L1 expression and clinicopathologic features in central-type and peripheral-type SCLC.

**Variable**		**PD-L1 expression in central-type SCLC**			**PD-L1 expression in peripheral-type SCLC**			
	**No. (%)**	**Positive No. (%)**	**Negative No. (%)**	***P*-value**	**No. (%)**	**Positive No. (%)**	**Negative No. (%)**	***P*-value**
Total	100	26 (26.0)	74 (74.0)		42	2 (4.8)	40 (95.2)	
Age (year)								
<60	51 (51.0)	15 (29.4)	36 (70.6)	0.498	16 (38.1)	1 (6.3)	15 (93.8)	
≥60	49 (49.0)	11 (22.4)	38 (77.6)		26 (61.9)	1 (3.8)	25 (96.2)	1.000
Sex								
Male	61 (61.0)	11 (18.0)	50 (82.0)	**0.035**	31 (73.8)	1 (3.2)	30 (96.8)	
Female	39 (39.0)	15 (38.5)	24 (61.5)		11 (26.2)	1 (9.1)	10 (90.9)	0.460
Smoking status								
Smoker	70 (70.0)	17 (24.3)	53 (75.7)	0.621	30 (71.4)	2 (6.7)	28 (93.3)	
Non-smoker	30 (30.0)	9 (30.0)	21 (70.0)		12 (28.6)	0 (0.0)	12 (100.0)	1.000
Stage								
Limited-stage	57 (57.0)	16 (28.1)	41 (71.9)	0.650	29 (69.0)	1 (3.4)	28 (96.6)	
Extensive-stage	43 (43.0)	10 (23.3)	33 (76.7)		13 (30.9)	1 (7.7)	12 (92.3)	0.528
Pleural invasion								
Present	8 (30.8)	2 (25.0)	6 (75.0)	0.420	5 (17.9)	0 (0.0)	5 (100.0)	
Absent	18 (69.2)	8 (44.4)	10 (55.6)		23 (82.1)	1 (4.3)	22 (95.7)	1.000
Vascular invasion								
Present	12 (46.2)	9 (75.0)	3 (25.0)	**0.001**	12 (42.9)	1 (8.3)	11 (91.7)	
Absent	14 (53.8)	1 (7.1)	13 (92.9)		16 (57.1)	0 (0.0)	16 (100.0)	0.429
Lymphatic invasion								
Present	13 (50.0)	9 (69.2)	4 (30.8)	**0.004**	7 (25.0)	0 (0.0)	7 (100.0)	
Absent	13 (50.0)	1 (7.7)	12 (92.3)		21 (75.0)	1 (4.8)	20 (95.2)	1.000
TTF-1 expression								
Positive	72 (72.0)	25 (34.7)	47 (65.3)	**0.001**	33 (78.6)	0 (0.0)	33 (100.0)	
Negative	28 (28.0)	1 (3.6)	27 (96.4)		9 (21.4)	2 (22.2)	7 (77.8)	**0.042**
Chromogranin A								
Positive	83 (84.7)	25 (30.1)	58 (69.9)	0.065	32 (76.2)	1 (3.1)	31 (96.9)	
Negative	15 (15.3)	1 (6.7)	14 (93.3)		10 (23.8)	1 (10.0)	9 (90.0)	0.424
Synaptophysin								
Positive	90 (91.8)	25 (27.8)	65 (72.2)	0.677	39 (92.9)	1 (2.6)	38 (97.4)	
Negative	8 (8.2)	1 (12.5)	7 (87.5)		3 (7.1)	1 (33.3)	2 (66.7)	0.139
CD56								
Positive	89 (92.7)	25 (28.1)	64 (71.9)	0.670	40 (95.2)	1 (2.5)	39 (97.5)	
Negative	7 (7.3)	1 (14.3)	6 (85.7)		2 (4.8)	1 (50.0)	1 (50.0)	0.094

*SCLC, small-cell lung cancer. The bold values indicates significantly difference*.

Significant differences in PD-L1 expression were observed between female and male sex (*p* = 0.035), and between central and peripheral SCLCs of the TTF-1 positive subtype. PD-L1 expression was positively correlated with the presence of vascular invasion (*p* = 0.003) and the presence of lymphatic invasion (*p* = 0.001) in TTF-1 positive SCLCs. Further, there were no significant correlations between PD-L1 expression and any of the clinicopathologic parameters in TTF-1 negative SCLCs (Table 3).

**Table 3:** Correlation between PD-L1 expression and clinicopathologic features in TTF-1-positive and TTF-1-negative SCLC.

	**PD-L1 expression in TTF-1-positive SCLC**				**PD-L1 expression in TTF-1-negative SCLC**			
**Variable**	**No. (%)**	**Positive No. (%)**	**Negative No. (%)**	***P*-value**	**No. (%)**	**Positive No. (%)**	**Negative No. (%)**	***P*-value**
Total	105	25 (23.8)	80 (76.2)		37	3 (8.1)	34 (91.9)	
Age (year)								
<60	50 (47.6)	15 (30.0)	35 (70.0)	0.175	17 (45.9)	1 (5.9)	16 (94.1)	
≥60	55 (52.4)	10 (18.2)	45 (81.8)		20 (54.1)	2 (10.0)	18 (90.0)	1.000
Sex								
Male	68 (64.8)	10 (14.7)	58 (85.3)	**0.004**	24 (64.9)	2 (8.3)	22 (91.7)	
Female	37 (35.2)	15 (40.5)	22 (59.5)		13 (35.1)	1 (7.7)	12 (92.3)	1.000
Smoking status								
Smoker	69 (65.7)	17 (24.6)	52 (75.4)	1.000	33 (89.2)	3 (9.1)	30 (90.9)	
Non-smoker	36 (34.3)	8 (22.2)	28 (77.8)		4 (10.8)	0 (0.0)	4 (100.0)	1.000
Tumor locations								
Central-type	72 (68.6)	25 (34.7)	47 (65.3)	**0.000**	28 (75.7)	1 (3.6)	27 (96.4)	
Peripheral-type	33 (31.4)	0 (0.0)	33 (100.0)		9 (24.3)	2 (22.2)	7 (77.8)	0.141
Stage								
Limited-stage	67 (63.8)	15 (22.4)	52 (77.6)	0.643	19 (51.4)	2 (10.5)	17 (89.5)	
Extensive-stage	38 (36.2)	10 (26.3)	28 (73.7)		18 (48.6)	1 (5.6)	17 (94.4)	1.000
Pleural invasion								
Present	13 (26.0)	2 (15.4)	11 (84.6)	0.688	33 (89.2)	2 (6.0)	31 (94.0)	
Absent	37 (74.0)	8 (21.6)	29 (78.4)		4 (10.8)	1 (25.0)	3 (75.0)	0.298
Vascular invasion								
Present	23 (46.0)	9 (39.1)	14 (60.9)	**0.003**	1 (25.0)	1 (100.0)	0 (0.0)	
Absent	27 (54.0)	1 (3.7)	26 (96.3)		3 (75.0)	0 (0.0)	3 (100.0)	0.250
Lymphatic invasion								
Present	20 (40.0)	9 (45.0)	11 (55.0)	**0.001**	33 (89.2)	2 (6.0)	31 (94.0)	
Absent	30 (60.0)	1 (3.3)	29 (96.7)		4 (10.8)	1 (25.0)	3 (75.0)	0.298
Chromogranin A								
Positive	94 (91.3)	25 (26.6)	69 (73.4)	0.109	21 (56.8)	1 (4.8)	20 (95.2)	
Negative	9 (8.7)	0 (0.0)	9 (100.0)		16 (43.2)	2 (12.5)	14 (87.5)	0.568
Synaptophysin								
Positive	102 (99.0)	25 (24.5)	77 (75.5)	1.000	27 (73.0)	1 (3.7)	26 (96.3)	
Negative	1 (1.0)	0 (0.0)	1 (100.0)		10 (27.0)	2 (20.0)	8 (80.0)	0.172
CD56								
Positive	97 (94.2)	24 (24.7)	73 (75.3)	1.000	32 (91.4)	2 (6.3)	30 (93.8)	
Negative	6 (5.8)	1 (16.7)	5 (83.3)		3 (8.6)	1 (33.3)	2 (66.7)	0.242

*SCLC, small-cell lung cancer. The bold values indicates significantly difference*.

We subsequently evaluated PD-L1 expression in small biopsy and surgically resected specimens (Table 4). Among the 88 small biopsy specimens, PD-L1 expression significantly correlated with TTF-1 (*p* = 0.023) and chromogranin A (*p* = 0.034) expression. In the 54 resected cases, PD-L1 expression was positively correlated with female sex (*p* = 0.010) and central location of the tumor (*p* = 0.002). Of the 54 resected SCLC, eight cases were also available as biopsy specimens for the comparison of PD-L1 expression between small biopsy and surgical resected specimens from the same patients. Of the eight paired cases, PD-L1 expression was observed in one case in which the biopsy and paired resected specimen showed the identical results with the similar intensity and percentage staining of the tumor cells (weak to moderate, 10% for resection and 8% for biopsy). The overall concordance rate of PD-L1 expression between resected and their corresponding biopsy specimens was 100%.

**Table 4:** Correlation between PD-L1 expression and clinicopathologic features in biopsy specimen and resection specimen of small-cell lung cancer.

	**PD-L1 expression in biopsy specimen**				**PD-L1 expression in resection specimen**			
**Variable**	**n (%)**	**Positive n (%)**	**Negative n (%)**	***P*-values**	**n (%)**	**Positiven n (%)**	**Negative n (%)**	***P*-values**
Total	88	17 (19.3)	71 (80.7)		54	11 (20.4)	43 (79.6)	
Age (year)								
<60	36 (40.9)	7 (19.4)	29 (80.6)	1.000	31 (57.4)	9 (29.0)	22 (71.0)	0.092
≥60	52 (59.1)	10 (19.2)	42 (80.8)		23 (42.6)	2 (8.7)	21 (91.3)	
Sex								
Male	57 (64.8)	9 (15.8)	48 (84.2)	0.272	35 (64.8)	3 (8.6)	32 (91.4)	0.010
Female	31 (35.2)	8 (25.8)	23 (74.2)		19 (35.2)	8 (42.1)	11 (57.9)	
Smoking status								
Smoker	66 (75.0)	14 (21.2)	52 (78.8)	0.545	34 (63.0)	5 (14.7)	29 (85.3)	0.294
Non-smoker	22 (25.0)	3 (13.6)	19 (86.4)		20 (37.0)	6 (30.0)	14 (70.0)	
Tumor locations								
Peripheral-type	74 (84.1)	16 (21.6)	58 (78.4)	0.288	26 (48.1)	10 (38.5)	16 (61.5)	0.002
Central-type	14 (15.9)	1 (7.1)	13 (92.9)		28 (51.9)	1 (3.6)	27 (96.4)	
Stage								
Limited-stage	38 (43.2)	7 (18.4)	31 (81.6)	1.000	50 (92.6)	11 (22.0)	39 (78.0)	0.571
Extensive-stage	50 (56.8)	10 (20.0)	40 (80.0)		4 (7.4)	0 (0.0)	4 (100.0)	
TTF-1 expression								
Positive	55 (62.5)	15 (27.3)	40 (72.7)	0.023	50 (92.6)	10 (20.0)	40 (80.0)	1.000
Negative	33 (37.5)	2 (6.1)	31 (93.9)		4 (7.4)	1 (25.0)	3 (75.0)	
Chromogranin A								
Positive	70 (81.4)	23 (32.9)	47 (67.1)	0.034	45 (83.3)	32 (71.1)	13 (28.9)	0.053
Negative	16 (18.6)	1 (6.3)	15 (93.8)		9 (16.7)	3 (33.3)	6 (66.7)	
Synaptophysin								
Positive	76 (88.4)	16 (21.1)	60 (78.9)	0.679	53 (98.1)	10 (18.9)	43 (81.1)	0.204
Negative	10 (11.6)	1 (10.0)	9 (90.0)		1 (1.9)	1 (100.0)	0 (0.0)	
CD56								
Positive	77 (91.7)	16 (20.8)	61 (79.2)	1.000	52 (96.3)	10 (19.2)	42 (80.8)	0.369
Negative	7 (8.3)	1 (14.3)	6 (85.7)		2 (3.7)	1 (50.0)	1 (50.0)	

### Survival Analysis

**Figure 2:**
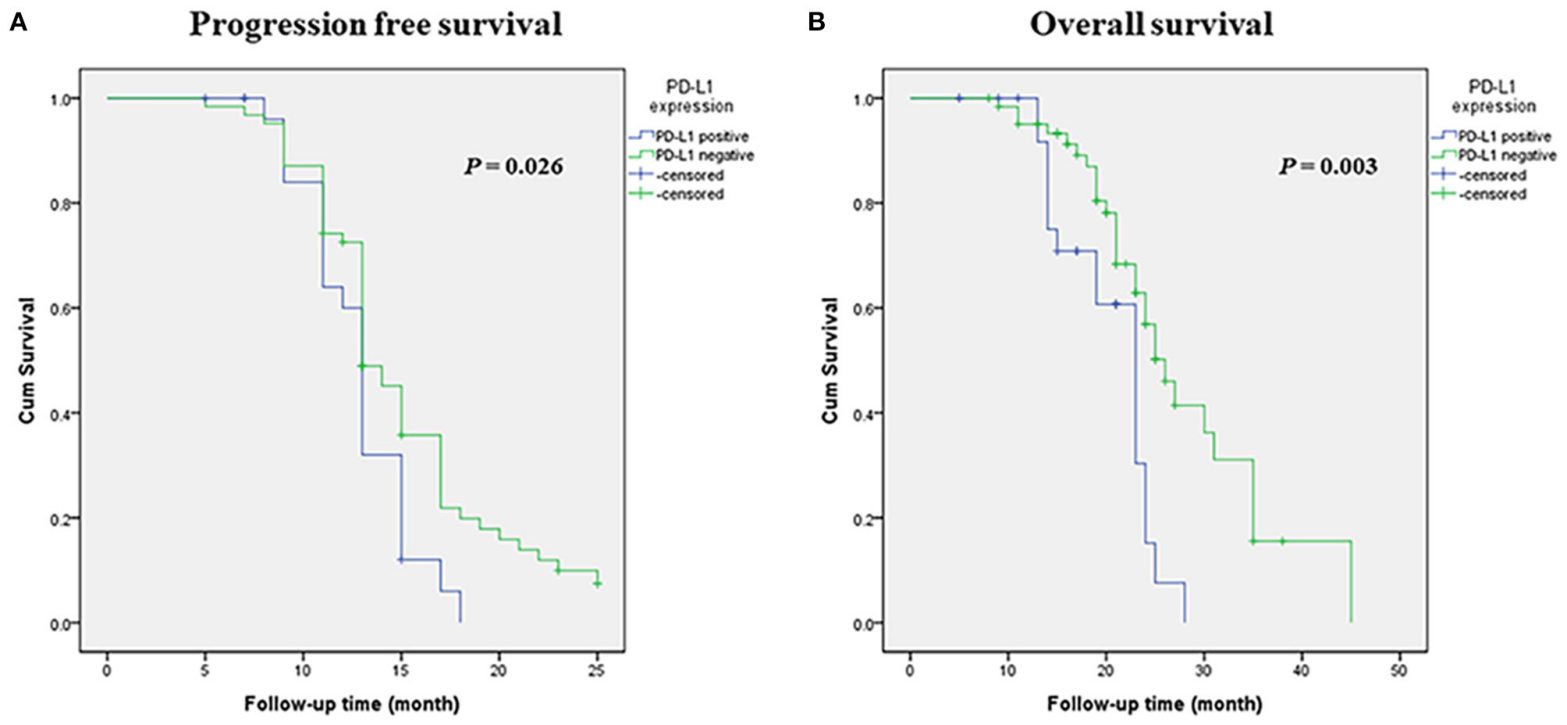
Kaplan-Meier survival estimates of progression-free survival (PFS) and overall survival (OS) based on the expression of PD-L1. **(A)** Kaplan-Meier plot showing PFS. **(B)** Kaplan-Meier plot showing OS.

Survival analysis was performed among the entire SCLC cohort and then among the different subtypes. In univariate analysis, PD-L1 expression was associated with a significantly poorer PFS and OS (*p* = 0.026 and *p* = 0.003, respectively) in entire SCLC cohort (Figures 2A,B). Multivariate Cox analysis confirmed that PD-L1 expression was an independent poor prognostic factor for OS (HR, 2.317; 95% CI 1.199–4.478; *p* = 0.012) and tended to be an independent prognostic factor for PFS (HR, 1.636; 95% CI 0.990–2.703; *p* = 0.051) (Table 5). In TTF-1 positive and central type SCLCs, PD-L1 expression was associated with poorer PFS (*p* = 0.014 and *p* = 0.042, respectively) and OS (*p* = 0.009 and *p* = 0.019, respectively), and in peripheral type SCLC, with poorer OS (*p* = 0.005).

**Table 5:** Univariate and multivariate analyses of factors associated with PFS and OS in small cell lung cancer patients.

**Factor**	**Category**	**PFS**					**OS**				
		**Mean**	**Univariate**		**Multivariate**		**Mean**	**Univariate**		**Multivariate**	
			***p***	**HR (95% CI)**	***p***	**HR (95% CI)**		***p***	**HR (95% CI)**	***p***	**HR (95% CI)**
TTF1 expression	Positive vs.	17.82 ± 0.54	0.311	1.259 (0.764–2.075)			29.35 ± 1.12	0.423	1.300 (0.662–2.552)		
	Negative	19.96 ± 0.65					32.81 ± 2.94				
Tumor types	Central vs.	19.25 ± 0.056	0.6	0.600 (0.376–0.956)			29.43 ± 1.19	0.501	0.783 (0.430–1.425)		
	Peripheral	19.51 ± 0.97					31.93 ± 2.46				
Vascular invasion	Present vs.	20.07 ± 0.81	0.808	1.066 (0.596–1.908)			30.96 ± 1.80	0.976	1.011 (1.464–2.204)		
	Absent	20.53 ± 0.91					32.49 ± 2.93				
Lymphatic invasion	Present vs.	19.55 ± 0.74	0.188	1.430 (0.782–2.613)			29.88 ± 1.81	0.377	1.392 (0.636–3.045)		
	Absent	21.45 ± 0.98					33.40 ± 3.03				
Pleural invasion	Present vs.	19.23 ± 1.16	0.343	1.344 (0.674–2.681)			28.15 ± 2.56	0.202	1.635 (0.715–3.741)		
	Absent	20.58 ± 0.74					31.92 ± 2.11				
PD-L1 expression	Positive vs.	17.82 ± 0.54	0.026	1.640 (0.994–2.707)	0.055	1.636 (0.990–2.703)	25.61 ± 1.04	0.003	2.514 (1.325–4.771)	0.012	2.317 (1.199–4.478)
	Negative	19.96 ± 0.65					32.87 ± 1.70				
Stage	Extensive vs.	18.08 ± 0.91	0.051	1.745 (1.060–2.875)	0.087	1.566 (0.937–2.616)	27.04 ± 1.96	0.072	1.020 (0.447–2.329)	0.265	1.450 (0.754–2.786)
	Limited	19.85 ± 0.58					31.77 ± 1.50				

## Discussion

To the best of our knowledge, this is the first study to investigate the correlation among tumor location, TTF-1 expression, and sample type and PD-L1 expression in SCLCs. In this study, the clinicopathologic and prognostic significance of PD-L1 expression were assessed in 142 cases of SCLC and different SCLC subsets. We demonstrated that PD-L1 expression was more frequent in females than males, in central type than peripheral type SCLCs, and in TTF-1 positive than negative SCLCs. PD-L1 expression was positively associated with vascular and lymphatic invasion, which have been implicated in unfavorable prognosis. PD-L1 expression was associated with a significantly poorer PFS and OS in all SCLCs as well as in TTF-1 positive and central type SCLCs. Multivariate analysis revealed that PD-L1 expression in SCLC was an independent prognostic factor for OS and tended to be an independent prognostic factor for PFS.

So far, there is no study on the relationship between the expression of PD-L1 and gender by using 22C3 antibody in patients with small cell lung cancer. There are some studies on the relationship between the expression of PD-L1 and gender in non-small-cell lung cancer (NSCLC). One of the result showed that there is no significant correlation between gender and the expression of PD-L1 (18). However, the other literature results showed that the expression rate of PD-L1 is higher in male patients (19). Some animal studies and emerging clinical evidence suggested a role for estrogens in upregulation of PD-1 and PD-L1 expression, this may be a factor in the high expression level of PD-L1 in women, but further research is needed to confirm the above results (20, 21).

Multiple clinical trials have defined PD-L1 as a biomarker for PD-1/PD-L1 checkpoint inhibitor treatment in NSCLC, and PD-L1 is expressed in ~50–70% of NSCLC as reported in the previous studie (22–25). However, the immunotherapeutic efficacy of PD-L1 in SCLC is far lower than that in NSCLC, and PD-L1 expression is relatively low in SCLC compared to NSCLC. Limited number of studies have evaluated PD-L1 expression in SCLC. PD-L1 expression was found in 19.7% of SCLCs in our study, and this result is consistent with two previous studies in which the expression rates were 18 and 25%, respectively (26, 27). Two other studies showed relatively lower rates of PD-L1 expression in SCLCs (2.5 and 10%), but the sample sizes in those studies were small (39 and 30, respectively) (28, 29). The reason of the variable expression in PD-L1 positivity in our study compared to the other studies may be the use of different types of antibodies as well as the variable definitions of PD-L1 positivity. However, Yasuda et al. (29) used the same antibody and same scoring criteria, they found positivity of only 2.5%. Except for the sample size, the heterogeneity of the study population and interpretation criteria may be responsible for the differences in PD-L1 expression levels. First of all, according to the interpretation criteria, PD-L1 protein expression is determined by using Tumor Proportion Score (TPS), which is the percentage of tumor cells showing partial or complete membrane staining with any extent (1+ - 3+). Specimens with a 1+ result in routine immunohistochemical staining are sometimes judged negative, but in the latest criteria, as long as there is a positive cell membrane, it should be judged as positive. All the samples we selected were not treated with chemotherapy, which can avoid the effect of chemotherapy on the expression level of PD-L1, which may also be a reason. Further large-scale investigations or multicenter studies are warranted to determine the actual rate of expression of PD-L1 in SCLCs. PD-L1 was identified as a poor prognostic factor in the current study. The prognostic role of PD-L1 in SCLC is still unclear due to the relatively small number of reports available and the different antibody clones used in the reports (14, 15, 30). Besides tumor cells, lymphocytes, and macrophages in the stroma were also included in the evaluation in several studies (26) which showed PD-L1 expression rates ranging between 31.7 and 45.7%, which were similar to the results from our study (41.5%, 59/142) (28, 31). In our study, PD-L1 expression on the inflammatory cells was also analyzed in terms of clinicopathological parameters and prognosis, but no positive correlations were noted.

Although the implementation of anti-PD-1/PD-L1 therapy in SCLC has improved the clinical outcome for a small percentage of patients, the majority of patients show little to no response to the treatment even in cases with high PD-L1 expression. Conversely, clinical benefit from immunotherapeutic agents has also been observed in patients with PD-L1-negative tumors (5, 31). These data support the notion that PD-L1 expression cannot be considered as a unique criterion to predict which population will benefit from anti-PD-1 antibodies. Therefore, identification of additional predictors is imperative to improve the response to immune checkpoint inhibitors. Association analysis between the expression of PD-L1 and clinicopathological factors demonstrated high frequency of PD-L1 expression in both central type and TTF-1 expressed SCLC. Primary tumor location has been reported to predict prognosis of SCLC (12, 32). A previous study using a mouse model of SCLC reported that centrally located tumors showed better response to cisplatin than peripheral lesions (7). Based on the differential expression of PD-L1, we speculate that peripheral and central SCLCs may show different therapeutic reactivity to immunotherapy. TTF-1 is a master regulator of lung morphogenesis and an important marker for SCLC. TTF-1 expression in SCLC has been reported to be associated with neuroendocrine differentiation and aggressive biology (8, 13). However, TTF-1 is not a marker specific for SCLCs, a significant proportion of extrapulmonary SCLCs are positive for TTF-1. In other studies, TTF-1 has been proposed to be a candidate lineage lung oncogene, knockdown of it reduced growth, and cell viability of lung cancer containing the TTF-1 amplicon (24). A study by Takahashi et al. also demonstrated that TTF-1 stimulated the AKT pathway by directly regulating the expression of a tyrosine kinase-like receptor (22), and AKT pathway has been reported to regulate the expression of PD-L1 in lung cancer (31). The high expression of PD-L1 in TTF-1 positive cases may due to activated oncogenic pathway which was mediated by TTF-1. The positive association between PD-L1 and TTF-1 expression has been reported in pulmonary sarcomatoid carcinomas (33). Furthermore, TTF-1 expression was shown to predict the response to immune checkpoint inhibitors in NSCLC (34). So it can be speculated that high TTF-1 expression could be a predictive marker for anti-PD-1/PD-L1 therapy in SCLC. Further studies are needed to elucidate this possibility and the underlying mechanisms.

Discordances between surgically resected and biopsy specimens have been reported in several studies in NSCLC (35, 36). However, there is no relevant research in SCLC at present, and this may be because most SCLC cases are unresectable, and only a small number of patients receive surgery owing to the lack of preoperative biopsy results. To investigate whether expressional heterogeneity of PD-L1 exists between biopsy and resection specimens, our study evaluated the PD-L1 expression in the two types of samples and revealed no heterogeneity of PD-L1 expression between the two type of samples. This may due to the relatively lower intratumoral (within a tumor) heterogeneity of SCLCs compared to NSCLCs.

The above findings provide the first evidence of an association between PD-L1 expression, central location, and TTF-1 expression in SCLC. Nevertheless, this study has a few limitations. Firstly, we only included those resected cases that had follow-up data and sufficient specimen, which may have introduced a bias into the analysis. Secondly, the data were collected retrospectively, including some of the information on smoking history which was obtained by telephonic enquiry. It is plausible that some of the patient families may not have provided particularly accurate information, which may explain why there were relatively more non-smokers in our cohort. Another possible reason is that indoor air pollution or secondhand smoking at home or at work may have contributed to the increased risk of developing lung cancer.

In conclusion, our results revealed a significant association of PD-L1 expression with female sex, central tumor location, TTF-1 expression, and prognosis of the patients with SCLC. Our study provides compelling evidences which can help guide future research and clinical trials in SCLC. However, further studies are needed before drawing definitive conclusions and implementing them clinically.

## Synopsis

This study retrospectively analyzed the effect of PD-L1 expression in different types of small-cell lung cancers (SCLCs), in terms of the clinicopathologic features and survival, with the objective of identifying more specific candidates for immunotherapy.

## Data Availability Statement

The original contributions presented in the study are included in the article/supplementary material, further inquiries can be directed to the corresponding author/s.

## Author Contributions

P-LS designed the review. SY collected the data and prepared the draft. YL and MJ participated in data interpretation. P-LS and HG provided research fund. All authors read and approved the final manuscript.

## Conflict of Interest

The authors declare that the research was conducted in the absence of any commercial or financial relationships that could be construed as a potential conflict of interest.
